# Supplementary material for: Epigenetic profiling of hematopoietic stem cells from male mice identifies KDR and PU.1 as regulators of aging transcriptome and caloric restriction response
Source: Nat Commun. 2026 Feb 20;17:2978. doi: 10.1038/s41467-026-69718-0 (PMC13035812; doi:10.1038/s41467-026-69718-0)
Supplement: Supplementary file 1 — Supplementary Information [file 41467_2026_69718_MOESM1_ESM.pdf]

Supplementary Fig. 1

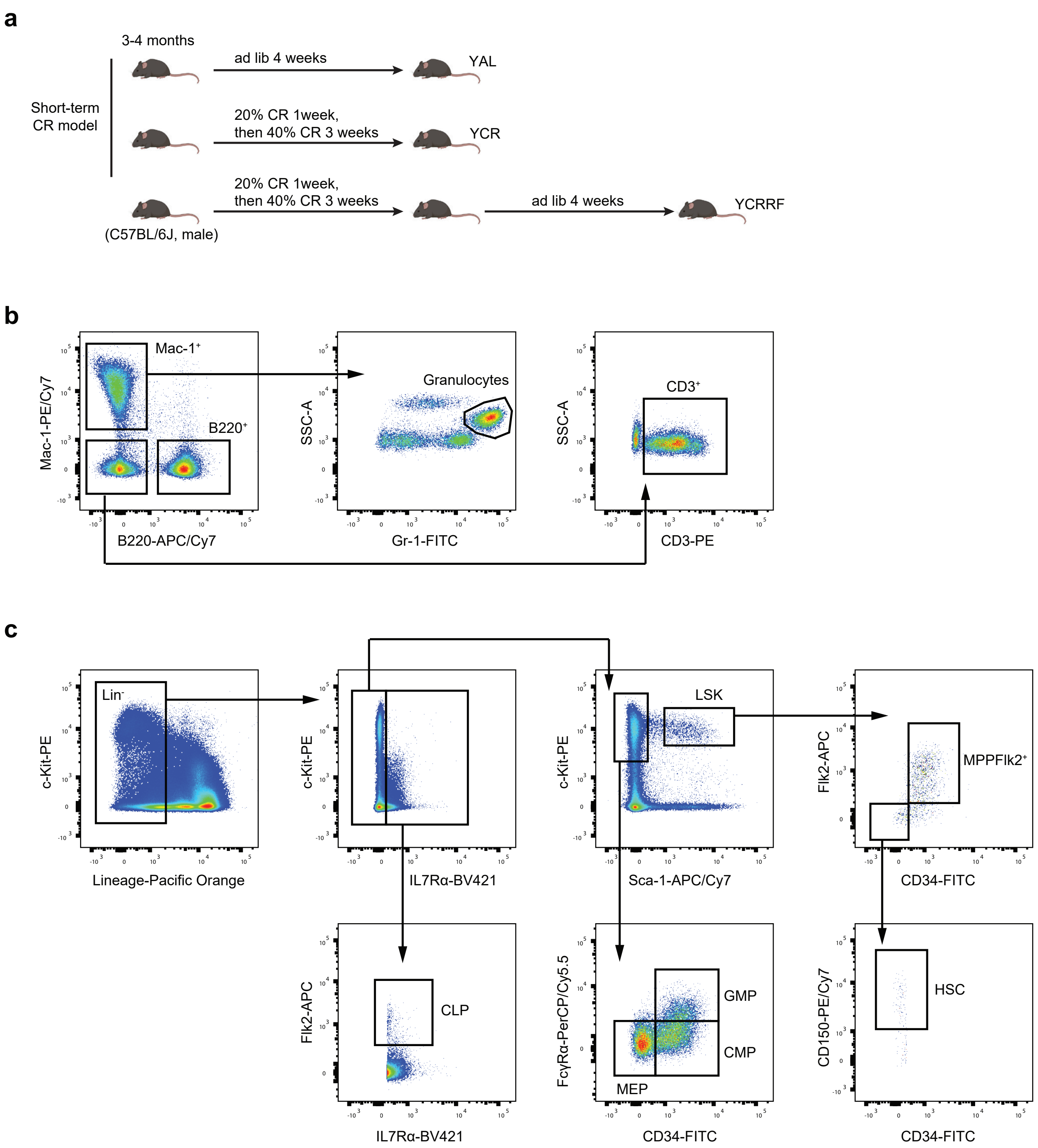

Supplementary Fig. 1: Short-term CR paradigms, and the gating strategy.

(**a**) Short-term CR paradigms. YAL (Young Ad Libitum), 4 weeks ad libitum feeding; YCR (Young Caloric Restricted), 4 weeks CR; YCRRF (Young Caloric Restricted Re-Fed), 4 weeks ad libitum access to food after 4 weeks CR. Created in BioRender. Ma, F. (2025) https://BioRender.com/fdntz9h.

(**b**) Gating strategy of peripheral blood (PB) analysis.

(**c**) Gating strategy of whole bone marrow (WBM) analysis.

Supplementary Fig. 2

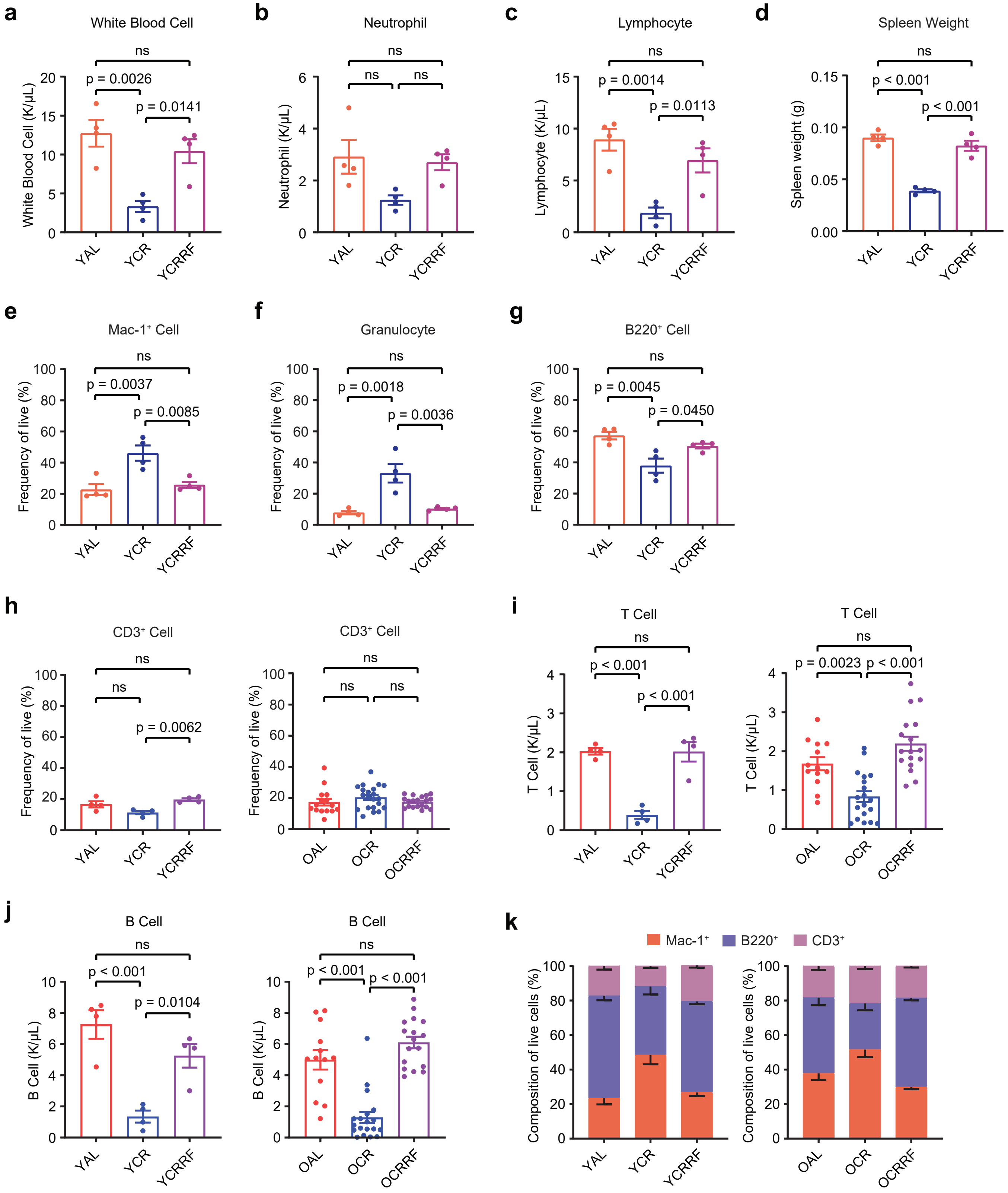

Supplementary Fig. 2: Complete blood count and FACS analysis of peripheral blood in short-term and lifelong CR mice.

(**a**-**c**) Complete blood count of white blood cells (**a**), neutrophils (**b**), and lymphocytes (**c**) in YAL, YCR, and YCRRF mice. Data are represented as mean ± SEM, n = 4, one-way ANOVA. Source data are provided as a Source Data file.

(**d**) Spleen weight of YAL, YCR and YCRRF mice. Data are represented as mean ± SEM, n = 4, one-way ANOVA. Source data are provided as a Source Data file.

(**e-g**) Frequency of Mac-1^{+} cells (**e**), granulocytes (**f**), and B220^{+} cells (**g**) in the PB of YAL, YCR, and YCRRF mice. Data are represented as mean ± SEM, n = 4, one-way ANOVA. Source data are provided as a Source Data file.

(**h**) Frequency of CD3^{+} cells in the PB of young and old CR mice. Data are represented as mean ± SEM, young (n =4), OAL (n = 15), OCR (n = 21), OCRRF (n = 19), one-way ANOVA. Source data are provided as a Source Data file.

(**i,j**) Absolute count of T cells (**i**) and B cells (**j**) in young and old CR mice. Data are represented as mean ± SEM, young (n =4), OAL (n = 13), OCR (n = 19), OCRRF (n = 17), one-way ANOVA. Source data are provided as a Source Data file.

(**k**) Lineage composition of the PB in young and old CR mice. Data are represented as mean ± SEM, young mice (n = 4), OAL (n = 15), OCR (n = 21), OCRRF (n = 19). Source data are provided as a Source Data file.

Supplementary Fig. 3

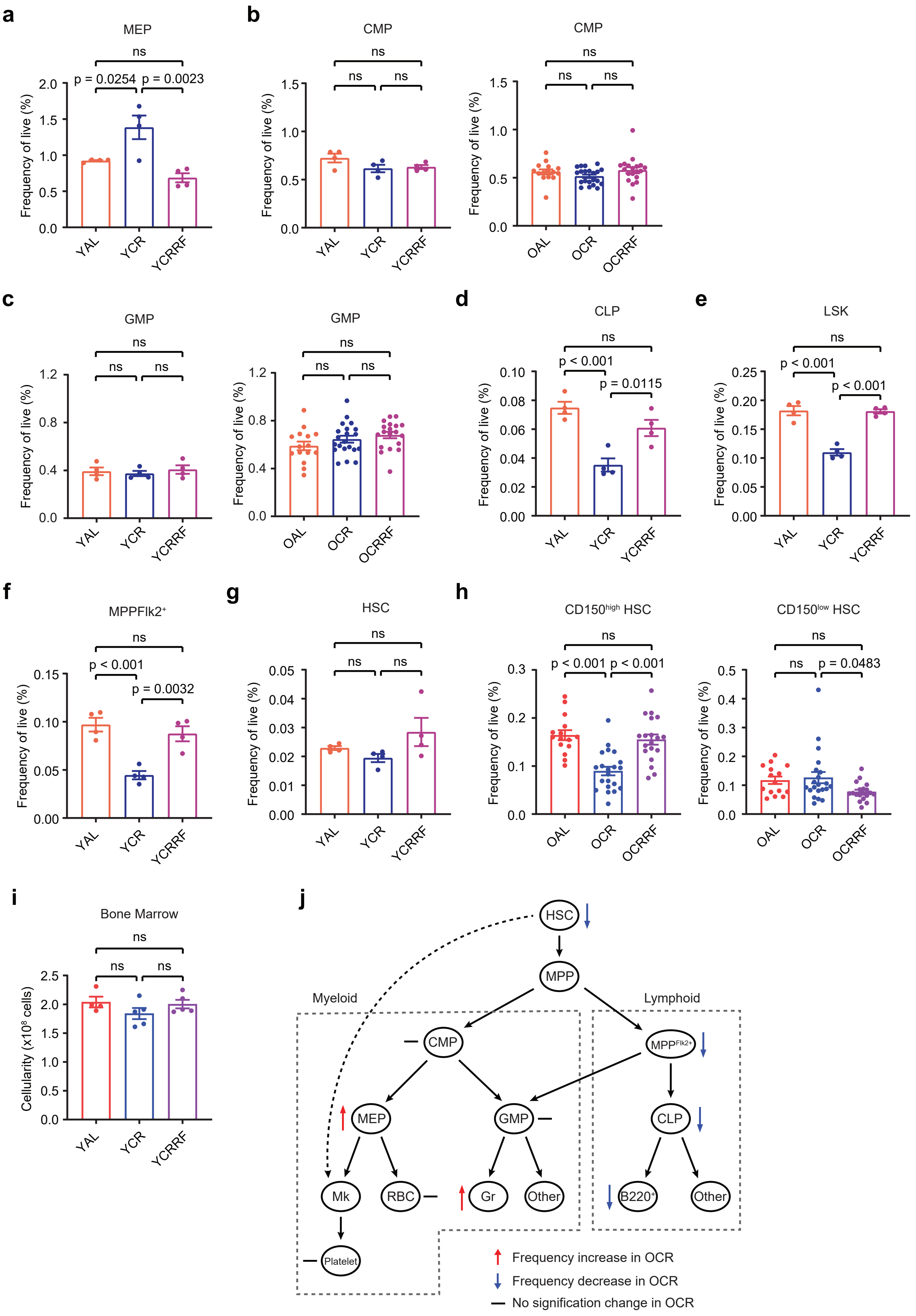

Supplementary Fig. 3: FACS analysis of the WBM in short-term and lifelong CR mice.

(**a**-**h**) Frequency of megakaryocyte-erythroid progenitors (MEP) (**a**), common myeloid progenitors (CMP) (**b**), granulocyte-monocyte progenitors (GMP) (**c**), common lymphoid progenitors (CLP) (**d**), $Lin^{-}Sca\text{-}1^{+}c\text{-}Kit^{+}$ cells (LSK) (**e**), lymphoid-biased multipotent progenitors ($MPPFlk2^{+}$) (**f**), and hematopoietic stem cells (HSCs) (**g**), $CD150^{high}$ and $CD150^{low}$ HSCs (**h**) in the bone marrow of young and/or old CR mice. Data are represented as mean ± SEM, young (n =4), OAL (n = 15), OCR (n = 21), OCRRF (n = 19), one-way ANOVA. Source data are provided as a Source Data file.

(**i**) Bone marrow cellularity in YAL, YCR, and YCRRF mice. Data are represented as mean ± SEM, one-way ANOVA. YAL (n = 4), YCR (n = 5), YCRRF (n = 5). Source data are provided as a Source Data file.

(**j**) Model of hematopoiesis under CR stress showing prioritized myeloid differentiation and repressed lymphoid differentiation.

Supplementary Fig. 4

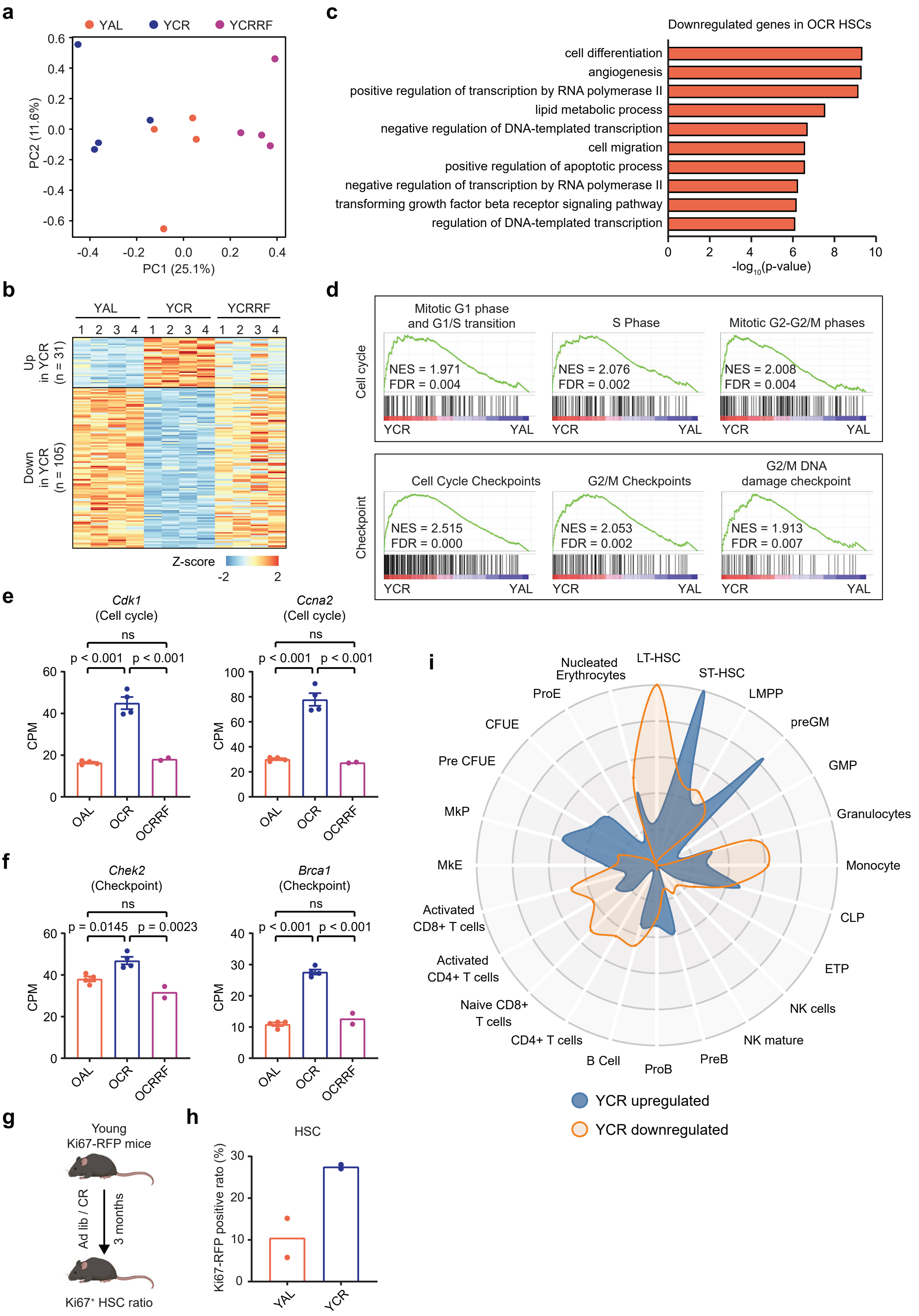

Supplementary Fig. 4: RNA-seq analysis of HSCs from short-term and lifelong CR mice.

(**a**) PCA plot of RNA-seq datasets from YAL (n = 4), YCR (n = 4), and YCRRF (n = 4) HSC samples.

(**b**) Heatmap of differentially expressed genes (DEGs) in YAL versus YCR comparison (FC > 1.2, FDR < 0.05). RNA-seq data of HSCs purified from YAL (n = 4), YCR (n = 4), and YCRRF (n = 4) mice were used.

(**c**) Pathway analysis of downregulated DEGs in OCR HSCs. Gene Ontology enrichment was performed using DAVID. Enrichment p-values correspond to the DAVID EASE Score (modified one-sided Fisher's exact test) without correction for multiple testing.

(**d**) GSEA analysis of cell cycle and checkpoint related pathways using RNA-seq data from YAL and YCR HSCs.

(**e-f**) Expression levels of *Cdk1* and *Ccna2* (E), *Chek2* and *Brca1* (F) in OAL (n = 4), OCR (n = 4), and OCRRF (n = 2) HSCs. Data are represented as mean ± SEM, one-way ANOVA.

(**g**) Short-term CR treatment in young Ki67-RFP mice. Ki67-RFP mice (3-4 months old) were subjected to 20% CR for the first week, followed by 40% CR for a total of 3 months. Ad lib fed mice were used as controls. Created in BioRender. Ma, F. (2025) https://BioRender.com/fdntz9h.

(**h**) Ratio of Ki67-RFP positive, cycling HSCs in ad lib and CR-treated Ki67-RFP mice. YAL (n = 2), YCR (n = 2). Source data are provided as a Source Data file.

(**i**) CellRadar plot derived from DEGs of YAL versus YCR comparison (FC > 1.2, FDR < 0.05). RNA-seq data of HSCs purified from YAL (n = 4), YCR (n = 4) mice were used.

Supplementary Fig. 5

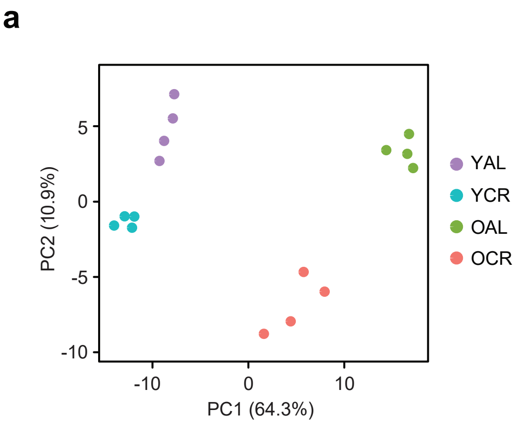

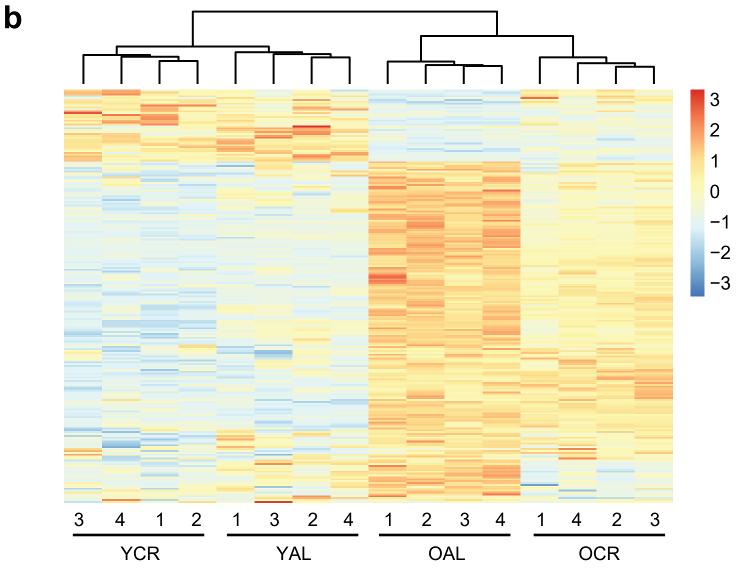

Supplementary Fig. 5: PCA and unsupervised clustering of RNA-seq data from HSCs of short-term and lifelong CR mice.

(**a**) PCA plot of RNA-seq data from HSCs of short-term and lifelong CR mice (n = 4).

(**b**) Unsupervised clustering of RNA-seq data from HSCs of short-term and lifelong CR mice (n = 4).

Supplementary Fig. 6

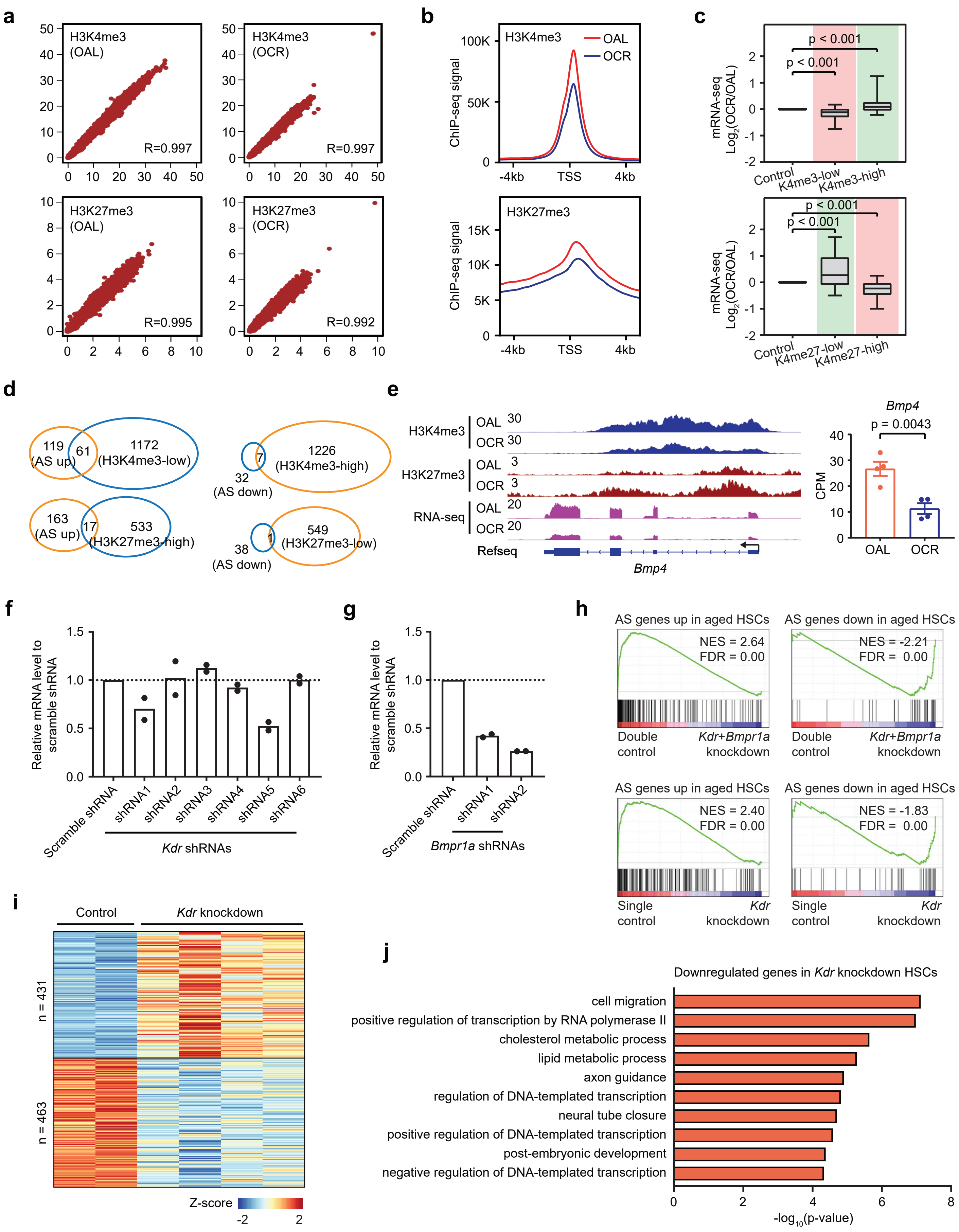

Supplementary Fig. 6: ChIP-seq analysis of H3K4me3 and H3K27me3 in OAL and OCR HSCs, along with the analysis of *Kdr* and/or *Bmpr1a* knockdown in aged HSCs.

(**a**) Scatter plots visualizing the correlation between two biological replicates of indicated histone modification ChIP-seqs generated with OAL or OCR HSCs. Pearson correlations are noted.

(**b**) Composite plots of H3K4me3 and H3K27me3 levels at the promoters of all genes. TSS, transcription start site.

(**c**) Box plots showing gene expression changes for genes categorized as H3K4me3-low and H3K4me3-high (upper) and H3K27me3-low and H3K27me3-high (lower) in OCR HSCs. RNA-seq data from OAL and OCR HSCs were used. Two-tailed t test. Box plots show the median (centre line), the 25th and 75th percentiles (box), and the 5th and 95th percentiles (whiskers).

(**d**) Venn diagram illustrating the overlap between AS genes and genes categorized as H3K4me3-high/low or H3K27me3-high/low in OCR HSCs.

(**e**) IGV tracks displaying H3K4me3, H3K27me3, and gene expression levels at the *Bmp4* loci. Expression levels of replicates were shown on the right. Data are represented as mean ± SEM, n = 4, two-tailed t test, p-value: < 0.01 **.

(**f-g**) Testing the knockdown efficiency of various shRNAs in vitro using HSCs purified from aged mice. HSCs were sorted into PVA-based media[40], then infected with shRNA viruses targeting *Kdr* (**f**) or *Bmpr1a* (**g**). GFP positive cells were sorted for RNA-seq 3.5 days post virus transduction.

(**h**) GSEA plots generated using AS genes. RNA-seq data from *Kdr* and *Bmpr1a* double knockdown (upper two panels) and *Kdr* single knockdown (lower two panels) HSCs were used. Double knockdown control, n = 2; double knockdown, n = 4; single knockdown control, n = 2; single knockdown, n = 4.

(**i**) Heatmap of DEGs in control versus *Kdr* knockdown comparison (FC > 1.2, p < 0.05). RNA-seq data of purified control (n = 2) or *Kdr* knockdown (n = 4) HSCs from recipient mice were used.

(**j**) Pathway analysis of downregulated DEGs in *Kdr* knockdown HSCs. Gene Ontology enrichment was performed using DAVID. Enrichment p-values correspond to the DAVID EASE Score (modified one-sided Fisher's exact test) without correction for multiple testing.

Supplementary Fig. 7

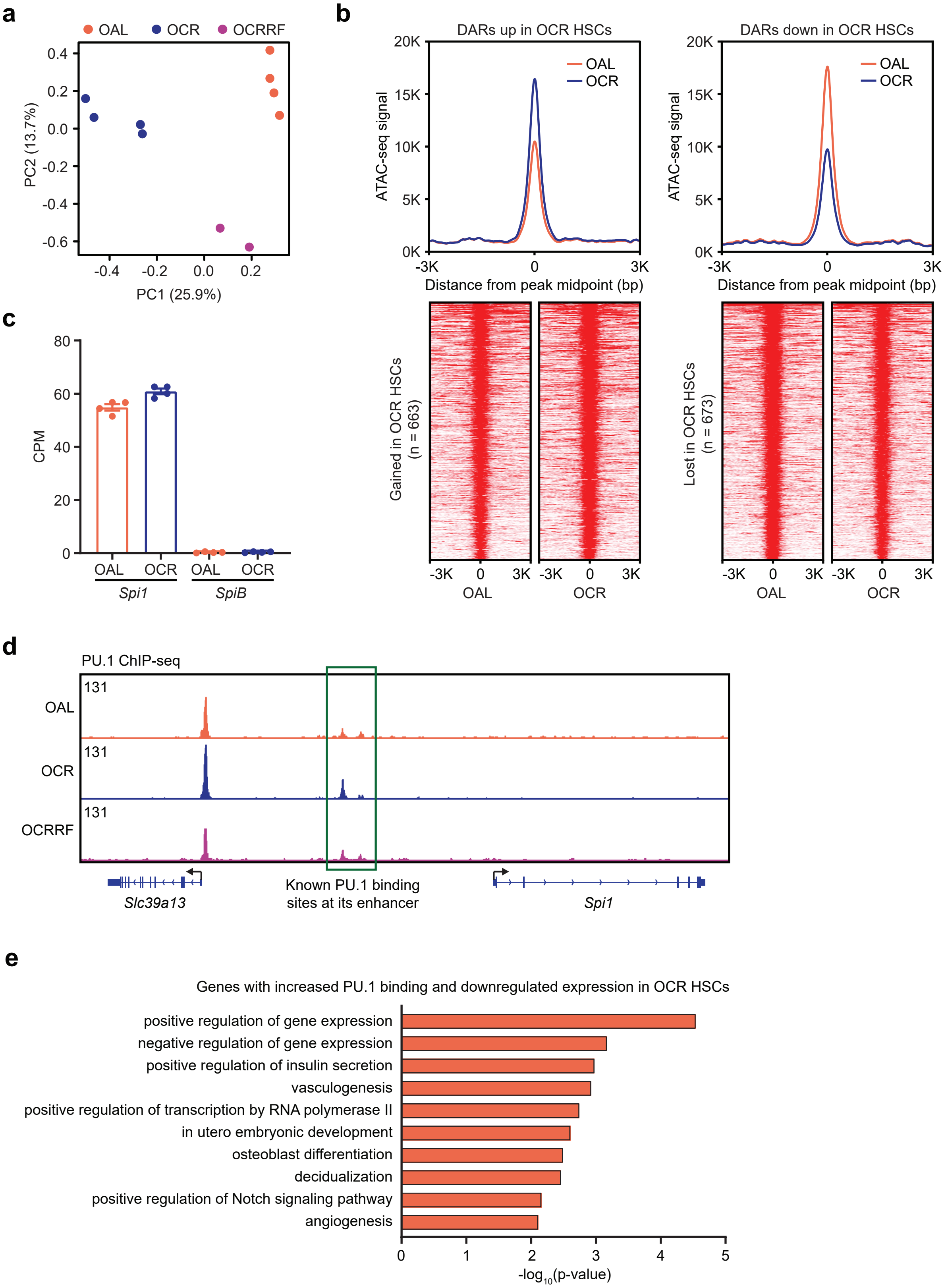

Supplementary Fig. 7: Chromatin accessibility and PU.1 ChIP-seq analysis in OAL, OCR and OCRRF HSCs.

(**a**) PCA plot of ATAC-seq datasets from OAL ($n = 4$), OCR ($n = 4$), and OCRRF ($n = 2$) HSC samples.

(**b**) Heatmap and composite plots of differentially accessible regions (DARs) between OAL and OCR HSCs ($FC > 1.5$, $FDR < 0.01$).

(**c**) Expression levels of *Spi1* and *SpiB* in OAL ($n = 4$) and OCR ($n = 4$) HSCs. Data are represented as mean ± SEM.

(**d**) IGV tracks displaying PU.1 signals at the *Spi1* loci.

(**e**) Pathway analysis of genes with increased PU.1 binding and downregulated expression in OCR HSCs. Gene Ontology enrichment was performed using DAVID. Enrichment p-values correspond to the DAVID EASE Score (modified one-sided Fisher's exact test) without correction for multiple testing.

Supplementary Fig. 8

a

Homer Known Motif Enrichment

PU.1 binding - up
Gene expression - up

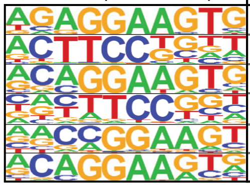

Transcription factor	p-value
PU.1	1 x e^{-60}
ELF4	1 x e^{-38}
ETS1	1 x e^{-34}
FLI1	1 x e^{-26}
GABPA	1 x e^{-25}
ERG	1 x e^{-24}

PU.1 binding - up
Gene expression - down

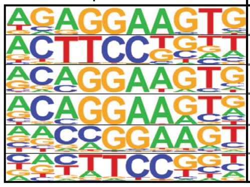

Transcription factor	p-value
PU.1	1 x e^{-43}
ELF4	1 x e^{-34}
ETS1	1 x e^{-23}
ERG	1 x e^{-20}
GABPA	1 x e^{-18}
FLI1	1 x e^{-17}

Homer *de novo* Motif Enrichment

PU.1 binding - up
Gene expression - up

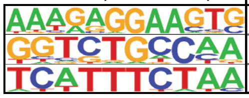

Transcription factor	p-value
PU.1, SPIB	1 x e^{-87}
NFIA	1 x e^{-9}
HMGA1	1 x e^{-8}

PU.1 binding - up
Gene expression - down

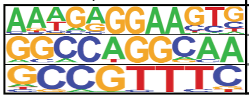

Transcription factor	p-value
PU.1, SPIB	1 x e^{-69}
ZFP335	1 x e^{-8}
MYBL2	1 x e^{-6}

b

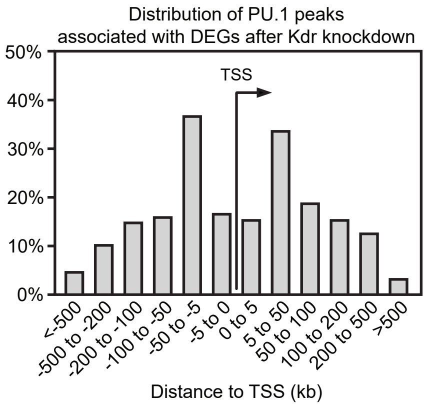

c

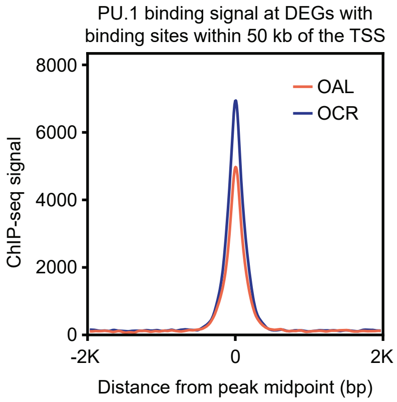

d

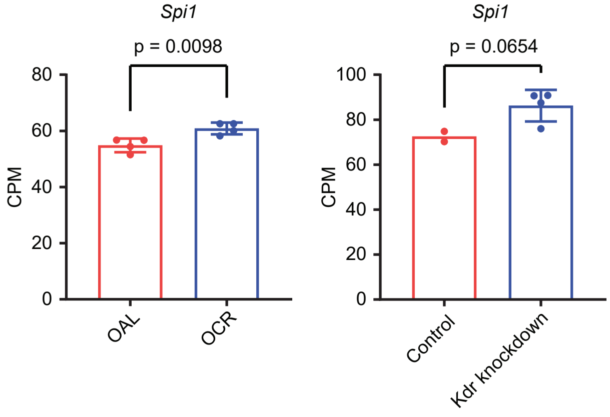

e

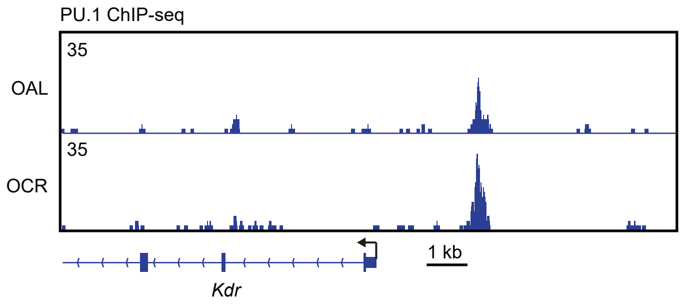

Supplementary Fig. 8: PU.1 motif enrichment and link to KDR signaling under CR.

(**a**) Transcription factor motif enrichment analysis of increased PU.1 peaks associated with genes upregulated or downregulated after CR. Both known (left) and *de novo* (right) motif analyses were performed using HOMER, examining sequences within ±200 bp of PU.1 peak centers.

(**b**) Distribution of increased PU.1 peaks associated with DEGs after *Kdr* knockdown.

(**c**) Composite plot of PU.1 ChIP-seq signals in OAL and OCR HSCs at *Kdr* knockdown associated DEGs with PU.1 binding sites within 50 kb of the TSS.

(**d**) *Spi1* expression in HSCs from lifelong CR, and *Kdr* knockdown mice. Data are represented as mean ± SEM, OAL and OCR (n = 4), control (n = 2), knockdown (n = 4), two-tailed t test.

(**e**) IGV tracks showing PU.1 occupancy near the *Kdr* promoter region.

Supplementary Fig. 9

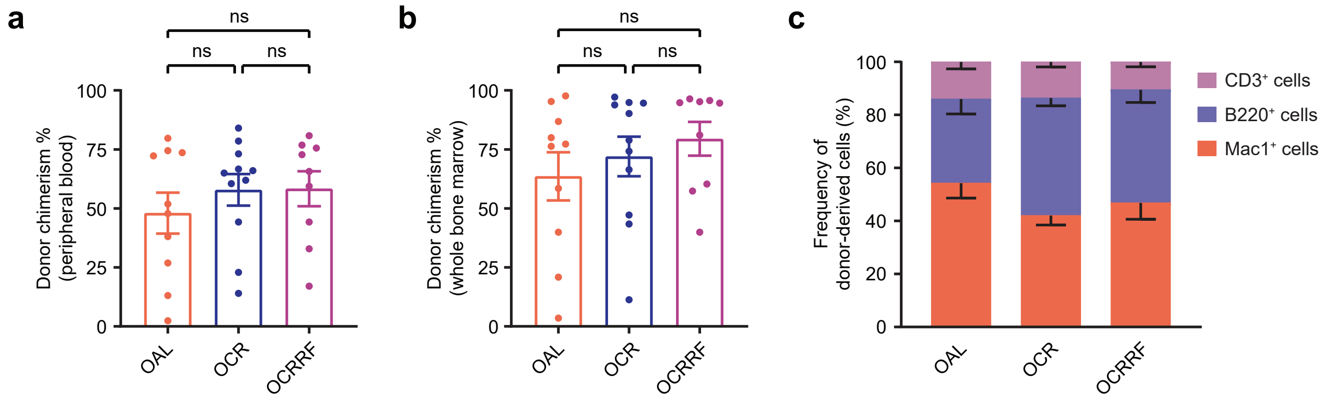

Supplementary Fig. 9: Donor chimerism and lineage output analysis of transplants with HSCs from OAL, OCR, or OCRRF mice.

(**a**-**b**) PB (**a**, 16 weeks) or WBM (**b**, 21 weeks) donor chimerism of recipient mice transplanted with HSCs purified from OAL, OCR, or OCRRF mice. 200 HSCs (CD45.2) were transplanted into lethally irradiated (9.56 Gy) recipients (CD45.1) together with $2x10^5$ WBM cells (CD45.1) via retro-orbital injection. Data are represented as mean ± SEM, OAL (n = 10), OCR (n = 11), OCRRF (n = 9), one-way ANOVA. Source data are provided as a Source Data file.

(**c**) Lineage composition of the PB (16 weeks) in recipient mice transplanted with HSCs purified from OAL, OCR, or OCRRF mice. Data are represented as mean ± SEM, OAL (n = 10), OCR (n = 11), OCRRF (n = 9). Source data are provided as a Source Data file.
