# Supplementary material for: Epigenetic profiling of hematopoietic stem cells from male mice identifies KDR and PU.1 as regulators of aging transcriptome and caloric restriction response
Source: Nat Commun. 2026 Feb 20;17:2978. doi: 10.1038/s41467-026-69718-0 (PMC13035812; doi:10.1038/s41467-026-69718-0)
Supplement: Supplementary file 2 — Description of Additional Supplementary Files [file 41467_2026_69718_MOESM2_ESM.pdf]

Description of Additional Supplementary Files

Supplementary Data 1: Genes with differential expression between age matched CR and AL HSCs

Differential expression was analyzed using DESeq2. Statistical significance was assessed using two-sided Wald tests, and p-values were adjusted for multiple testing using the Benjamini–Hochberg false discovery rate (FDR) method. Significantly changed genes were defined as those with an absolute fold change > 1.2 and FDR < 0.05. RNA-seq data of HSCs purified from YAL (n = 4), YCR (n = 4), OAL (n = 4) and OCR (n = 4) mice were used.

Supplementary Data 2-3: Expression changes of AS genes (Supplementary Data 2), Sirtuin gene and histone regulator (Supplementary Data 3) after lifelong CR

Differential expression was analyzed using DESeq2. Statistical significance was assessed using two-sided Wald tests, and p-values were adjusted for multiple testing using the Benjamini–Hochberg false discovery rate (FDR) method. Genes with FDR < 0.05 were considered significant. RNA-seq data of HSCs purified from OAL (n = 4) and OCR (n = 4) mice were used.
