# Supplementary material for: Epigenetic profiling of hematopoietic stem cells from male mice identifies KDR and PU.1 as regulators of aging transcriptome and caloric restriction response
Source: Nat Commun. 2026 Feb 20;17:2978. doi: 10.1038/s41467-026-69718-0 (PMC13035812; doi:10.1038/s41467-026-69718-0)
Supplement: Supplementary file 6 — Reporting Summary [file 41467_2026_69718_MOESM6_ESM.pdf]

nature portfolio

Corresponding author(s): Isabel Beerman

Last updated by author(s): Dec 10, 2025

Reporting Summary

Nature Portfolio wishes to improve the reproducibility of the work that we publish. This form provides structure for consistency and transparency in reporting. For further information on Nature Portfolio policies, see our Editorial Policies and the Editorial Policy Checklist.

Statistics

For all statistical analyses, confirm that the following items are present in the figure legend, table legend, main text, or Methods section.

n/a	Confirmed	
☐	☒	The exact sample size (*n*) for each experimental group/condition, given as a discrete number and unit of measurement
☐	☒	A statement on whether measurements were taken from distinct samples or whether the same sample was measured repeatedly
☐	☒	The statistical test(s) used AND whether they are one- or two-sided<br>*Only common tests should be described solely by name; describe more complex techniques in the Methods section.*
☐	☒	A description of all covariates tested
☐	☒	A description of any assumptions or corrections, such as tests of normality and adjustment for multiple comparisons
☐	☒	A full description of the statistical parameters including central tendency (e.g. means) or other basic estimates (e.g. regression coefficient) AND variation (e.g. standard deviation) or associated estimates of uncertainty (e.g. confidence intervals)
☐	☒	For null hypothesis testing, the test statistic (e.g. *F*, *t*, *r*) with confidence intervals, effect sizes, degrees of freedom and *P* value noted<br>*Give P values as exact values whenever suitable.*
☒	☐	For Bayesian analysis, information on the choice of priors and Markov chain Monte Carlo settings
☒	☐	For hierarchical and complex designs, identification of the appropriate level for tests and full reporting of outcomes
☐	☒	Estimates of effect sizes (e.g. Cohen's *d*, Pearson's *r*), indicating how they were calculated

Our web collection on statistics for biologists contains articles on many of the points above.

Software and code

Policy information about availability of computer code

Data collection	FACS data was collected on BD FACS Aria II, Fusion using Diva software. ATAC-seq, ChIP-seq and RNA-seq libraries were sequenced on Illunima HiSeq 2500 and NovaSeq 6000 System.
Data analysis	FACS Analysis was performed in FlowJo v10 and statistics data was generated in GraphPad Prism 9.<br><br>RNA-seq analysis<br>For analysis of transcriptome datasets, we built an index for STAR using the GENCODE M22 reference feature including protein-coding and non-coding genes. Prior to sequence alignment, we applied trim galore (version 0.4.3) with cutadapt (version 1.12) to remove any unnecessary genomic fragments (e.g. adapter dimers) and low-quality nucleotide sequences from the raw reads. We mapped adapter trimmed sequencing reads to the mouse reference genome (mm10) using STAR aligner and calculated the raw count using featureCounts software (gene-level). Differentially expressed gene (DEG) lists were generated with DESeq2. For transposable element (TE) detection, we utilized SQuIRE and used limma based edgeR package of R to find differential transposable elements.<br><br>ATAC-seq analysis<br>All sequencing reads were trimmed using cutadapt, and trimmed reads (>36 bp minimum alignment length) were mapped against the mm10 genome using BWA aligner. We used de-duplicated and uniquely mapped reads for peak calling analysis after excluding high-sensitive black-list regions defined by ENCODE. The candidate peaks were predicted by MACS peak calling tool (FDR < 0.01). After identifying narrow peaks from all replicates, we created a merged set of consensus peaks and generated a matrix of open chromatin regions (OCRs). This OCR matrix was then imported into the R package DESeq2, and we determined differentially accessible regions (DARs) with cutoff: FC > 1.5, logCPM > 1.5, FDR < 0.05. Finally, the candidate differential open chromatin regions were submitted to search for potential transcription factor binding sites using HOMER software with non-DARs as background regions. In this analysis, de novo motif and known motif searches were performed, and

we reported the top five significant de novo motif results. Composite plots and heatmaps were generated by customized Python script and Java TreeView software.

ChIP-seq analysis
All sequencing reads were trimmed using cutadapt, and trimmed reads (>36 bp minimum alignment length) were mapped against the mm10 genome using BWA aligner. We used de-duplicated and uniquely mapped reads for peak calling analysis after excluding high-sensitive black-list regions defined by ENCODE. For H3K4me3 ChIP-seq, signals within ±1 kb of the transcription start site (TSS) were used for downstream analysis. For H3K27me3 ChIP-seq, signals from 0 to 2 kb downstream of the TSS were used for further analysis. For PU.1 ChIP-seq, the candidate peaks were predicted by MACS peak calling tool (FDR < 0.05). Composite plots and heatmaps were generated with customized Python script and Java TreeView software.

Code availability
All customized Python scripts used in the manuscript are available via GitHub repository URL (https://github.com/genomicspark/ESCA_Unit_Scripts) and has been archived in Zenodo for citation (https://doi.org/10.5281/zenodo.17857712).

For manuscripts utilizing custom algorithms or software that are central to the research but not yet described in published literature, software must be made available to editors and reviewers. We strongly encourage code deposition in a community repository (e.g. GitHub). See the Nature Portfolio guidelines for submitting code & software for further information.

Data

Policy information about availability of data

All manuscripts must include a data availability statement. This statement should provide the following information, where applicable:

- Accession codes, unique identifiers, or web links for publicly available datasets
- A description of any restrictions on data availability
- For clinical datasets or third party data, please ensure that the statement adheres to our policy

All raw data are available at the NCBI Gene Expression Omnibus database (GEO; http://www.ncbi.nlm.nih.gov/geo/) under the accession number GSE284988 (https://www.ncbi.nlm.nih.gov/geo/query/acc.cgi?acc=GSE284988).

Research involving human participants, their data, or biological material

Policy information about studies with human participants or human data. See also policy information about sex, gender (identity/presentation), and sexual orientation and race, ethnicity and racism.

Reporting on sex and gender	N/A
Reporting on race, ethnicity, or other socially relevant groupings	N/A
Population characteristics	N/A
Recruitment	N/A
Ethics oversight	N/A

Note that full information on the approval of the study protocol must also be provided in the manuscript.

Field-specific reporting

Please select the one below that is the best fit for your research. If you are not sure, read the appropriate sections before making your selection.

☒ Life sciences ☐ Behavioural & social sciences ☐ Ecological, evolutionary & environmental sciences

For a reference copy of the document with all sections, see nature.com/documents/nr-reporting-summary-flat.pdf

Life sciences study design

All studies must disclose on these points even when the disclosure is negative.

Sample size	We used power calculations from previous experiments to determine the sample size.
Data exclusions	No data were excluded from the analyses.
Replication	Biological replicates of each experiments were described in the corresponding figure legends.
Randomization	The mice used in this study were randomly allocated to different experiment group.

Blinding	The investigators were blinded to group allocation during data collection and analysis.

Reporting for specific materials, systems and methods

We require information from authors about some types of materials, experimental systems and methods used in many studies. Here, indicate whether each material, system or method listed is relevant to your study. If you are not sure if a list item applies to your research, read the appropriate section before selecting a response.

Materials & experimental systems

n/a	Involved in the study	
☐	☒	Antibodies
☒	☐	Eukaryotic cell lines
☒	☐	Palaeontology and archaeology
☐	☒	Animals and other organisms
☒	☐	Clinical data
☒	☐	Dual use research of concern
☒	☐	Plants

Methods

n/a	Involved in the study	
☐	☒	ChIP-seq
☐	☒	Flow cytometry
☒	☐	MRI-based neuroimaging

Antibodies

Antibodies used	Flow cytometry. The following antibodies are from Biolegend: Biotin anti-TER119 (Cat# 116204, 1:100 dilution), Biotin anti-B220 (Cat# 103204, 1:100 dilution), Biotin anti-CD3 (Cat# 100244, 1:100 dilution), Biotin anti-Mac-1 (Cat# 101204, 1:100 dilution), Biotin anti-Gr-1 (Cat# 108404, 1:100 dilution), PB anti-TER119 (Cat# 116232, 1:200 dilution), PB anti-B220 (Cat# 103227, 1:200 dilution), PB anti-Mac-1 (Cat# 101224, 1:200 dilution), PB anti-CD3 (Cat# 100214, 1:200 dilution), BV421 anti-Gr-1 (Cat# 108445, 1:200 dilution), BV421 anti-IL7Rα (Cat# 135027, 1:200 dilution), APC/Cy7 anti-Sca-1 (Cat# 108126, 1:200 dilution), PE anti-c-Kit (Cat# 105808, 1:200 dilution), BV421 anti-c-Kit (Cat# 105827, 1:200 dilution), APC anti-Flk2 (Cat# 135310, 1:50 dilution), PE/Cy7 anti-CD150 (Cat# 115914, 1:200 dilution), APC anti-CD48 (Cat# 103412, 1:200 dilution), APC anti-CD45.1 (Cat# 110714, 1:200 dilution), PerCP/Cy5.5 anti-CD45.1 (Cat# 110728, 1:100 dilution), PB anti-CD45.2 (Cat# 109820, 1:100 dilution), PerCP/Cy5.5 anti-TER119 (Cat# 116228, 1:200 dilution), APC/Cy7 anti-B220 (Cat# 103224, 1:200 dilution), PE anti-CD3 (Cat# 100206, 1:200 dilution), PE/Cy7 anti-Mac-1 (Cat# 101216, 1:200 dilution), FITC anti-Gr-1 (Cat# 108406, 1:200 dilution), BV510 anti-Gr-1 (Cat# 108457, 1:200 dilution). FITC anti-CD34 (Cat# 11-0341-85, 1:50 dilution) and PerCP/Cy5.5 anti-FcγRα (Cat# 45-0161-82, 1:100 dilution) are from ThermoFischer.<br><br>ChIP-seq. Anti-H3K4me3 (Sigma, Cat# 07-473, 1.5 ul per IP), anti-H3K27me3 (Diagenode, Cat# C15410195, 1.5 ul per IP), anti- PU.1 (ThermoFischer, Cat# MA5-15064, 9 ul per IP), anti- PU.1 (abcam, Cat# ab227835, 9 ul per IP).
Validation	All antibodies used in this study are commercially available, and their validation is supported by references provided on the manufacturer's website.

Animals and other research organisms

Policy information about studies involving animals; ARRIVE guidelines recommended for reporting animal research, and Sex and Gender in Research

Laboratory animals	Life-long ad lib feeding and CR mice (male, C57BL/6, 24-26 months) were acquired from the NIA Aged Rodent Colony. Young C57BL/6 (CD45.2) male mice (3-4 months), Ki67-RFP reporter mice, and young female transplant recipient B6.SJL-Ptprca Pepcb/BoyJ (CD45.1) mice were obtained from The Jackson Laboratory. Mice were maintained under a 12 h light/12 h dark cycle, at an ambient temperature of 69–75 °F, with relative humidity of 30–70%.
Wild animals	No wild animals were involved in this study.
Reporting on sex	Male C57BL/6J mice were used in this study. For recipient B6.SJL-Ptprca Pepcb/BoyJ mice, female mice were used.
Field-collected samples	This study did not involve samples collected from the field.
Ethics oversight	Institutional Animal Care and Use Committees (National Institute on Aging)

Note that full information on the approval of the study protocol must also be provided in the manuscript.

Plants

Seed stocks

Report on the source of all seed stocks or other plant material used. If applicable, state the seed stock centre and catalogue number. If plant specimens were collected from the field, describe the collection location, date and sampling procedures.

Novel plant genotypes

Describe the methods by which all novel plant genotypes were produced. This includes those generated by transgenic approaches, gene editing, chemical/radiation-based mutagenesis and hybridization. For transgenic lines, describe the transformation method, the number of independent lines analyzed and the generation upon which experiments were performed. For gene-edited lines, describe the editor used, the endogenous sequence targeted for editing, the targeting guide RNA sequence (if applicable) and how the editor was applied.

Authentication

Describe any authentication procedures for each seed stock used or novel genotype generated. Describe any experiments used to assess the effect of a mutation and, where applicable, how potential secondary effects (e.g. second site T-DNA insertions, mosiacism, off-target gene editing) were examined.

ChIP-seq

Data deposition

☒ Confirm that both raw and final processed data have been deposited in a public database such as GEO.

☒ Confirm that you have deposited or provided access to graph files (e.g. BED files) for the called peaks.

Data access links
May remain private before publication.

ChIP-seq data generated by this study have been deposited to GEO with public accession of GSE284988.

Files in database submission

Provide a list of all files available in the database submission.

Genome browser session
(e.g. UCSC)

Provide a link to an anonymized genome browser session for "Initial submission" and "Revised version" documents only, to enable peer review. Write "no longer applicable" for "Final submission" documents.

Methodology

Replicates

ChIP-seq in this study were performed with two replicates.

Sequencing depth

Pair-end sequenced ChIP-seq samples (2x51bp for H3K9me3, 2x75bp for other marks):
samples total mapped uniq_read dedup_uniq_read
ChIP_OAL_H3K4me3_Rep1 101093753 89052961 78739963 36957478
ChIP_OAL_H3K4me3_Rep2 105281872 96365530 85737250 38287372
ChIP_OAL_H3K27me3_Rep1 95677805 91248261 82343754 56148091
ChIP_OAL_H3K27me3_Rep2 111487542 107116939 96984942 60116481
ChIP_OCR_H3K4me3_rep1 93108675 89374944 78072528 44762441
ChIP_OCR_H3K4me3_rep2 93465673 90310138 78797734 43731213
ChIP_OCR_H3K27me3_rep1 96566279 93293725 83946865 61206193
ChIP_OCR_H3K27me3_rep2 109238408 105528368 95252869 64250299
ChIP_OAL_PU.1_Rep1 83888049 8055354 7038871 5641755
ChIP_OAL_PU.1_Rep2 90670164 9303875 8119553 5983255
ChIP_OCR_PU.1_Rep1 81782920 8558860 7496878 5500357
ChIP_OCR_PU.1_Rep2 85980375 7967398 6966261 5412741
ChIP_OCRRF_PU.1_Rep1 89782745 7724527 6730549 5323981
ChIP_OCRRF_PU.1_Rep2 85543890 6320330 5504861 4418867

Antibodies

Anti-H3K4me3 (Sigma, Cat# 07-473), anti-H3K27me3 (Diagenode, Cat# C15410195), anti- PU.1 (ThermoFischer, Cat# MA5-15064), anti- PU.1 (abcam, Cat# ab227835).

Peak calling parameters

All sequencing reads were trimmed using cutadapt, and trimmed reads (>36 bp minimum alignment length) were mapped against an mm10 genome using BWA aligner. We used de-duplicated and uniquely mapped reads for peak calling analysis after excluding high-sensitive black-list regions defined by ENCODE. For H3K4me3, the narrow candidate peaks were predicted by MACS peak calling software (FDR < 0.05, shift 0, extsize 150). The sequencing libraries of all the histone marks were normalized (10 M reads). These normalized files were used to perform broad peak calling and chromatin state analysis. For H3K27me3, we identified broad peaks from IDEAS chromatin states (FDR < 0.05) and expanded the peak range to include any markers that appeared significant compared to random background height (average broad peak height > 0.3, and significant p-val < 1e-4). PU.1 peak calling was performed using MACS2 (FDR<0.05).

Data quality

Strong peaks were confirmed with visualization through genome browser for all samples.

Software

BWA-MEM (v.2.2.1): https://github.com/lh3/bwa
IDEAS (v.1.0.0): https://github.com/seqcode/IDEAS
MACS2 (v.2.2.7.1): https://pypi.org/project/MACS2

Flow Cytometry

Plots

Confirm that:

☒ The axis labels state the marker and fluorochrome used (e.g. CD4-FITC).

☒ The axis scales are clearly visible. Include numbers along axes only for bottom left plot of group (a 'group' is an analysis of identical markers).

☒ All plots are contour plots with outliers or pseudocolor plots.

☒ A numerical value for number of cells or percentage (with statistics) is provided.

Methodology

Sample preparation	For peripheral blood sample preparation, blood samples were treated with ACK twice to remove red blood cells, then stained with antibody cocktail.<br>For whole bone marrow sample preparation, bones from legs and/or arms were crushed and filtered. Then bone marrow cells were treated with ACK once to remove red blood cells then stained with antibody cocktail.<br>For c-kit enrichment and cell sorting, bone marrow cells were positive selected using PE-c-kit antibody and EasySep™ PE Positive Selection Kit II, then stained with antibody cocktail.
Instrument	BD FACS Aria II, BD FACS Fusion.
Software	BD FACSDiva and FlowJo v10 software were used for data acquisition and analysis.
Cell population abundance	Cell purity was assessed after sorting, with more than 98% purity achieved after each sorting.
Gating strategy	Included in Extended data Figure 1.

☒ Tick this box to confirm that a figure exemplifying the gating strategy is provided in the Supplementary Information.
